# An overview of realist evaluation for simulation-based education

**DOI:** 10.1186/s41077-018-0073-6

**Published:** 2018-07-17

**Authors:** Alastair C Graham, Sean McAleer

**Affiliations:** 1 Montagu Clinical Simulation Centre, Doncaster and Bassetlaw Teaching Hospitals NHS Foundation Trust, Montagu Hospital, Adwick Road, Mexborough, S64 0AZ SYK England; 2 Centre for Medical Education, The Mackenzie Building, Kirsty Semple Way, Dundee, DD2 4BF Scotland

**Keywords:** Simulation, Simulation-based education, Realistic evaluation, Realist evaluation, Realism, Medical students

## Abstract

This article describes the key features of realist (realistic) evaluation and illustrates their application using, as an example, a simulation-based course for final year medical students. The use of simulation-based education (SBE) is increasing and so too is the evidence supporting its value as a powerful technique which can lead to substantial educational benefits. Accompanying these changes is a call for research into its use to be more theory-driven and to investigate both ‘Did it work?’ and as importantly ‘Why did it work (or not)?’ An evaluation methodology that is capable of answering both questions is realist evaluation.

Realist evaluation is an emerging methodology that is suited to evaluating complex interventions such as SBE. The realist philosophy positions itself between positivist and constructivist paradigms and seeks to answer the question ‘What works for whom, in what circumstances and why?’ In seeking to answer this question, realist evaluation sets out to identify three fundamental components of an intervention, namely context, mechanism and outcome. Educational programmes work (successful outcomes) when theory-driven interventions (mechanisms) are applied to groups under appropriate conditions (context). Realist research uses a mixed methods (qualitative and quantitative) approach to gathering data in order to test the proposed context-mechanism-outcome (CMO) configurations of the intervention under investigation.

Realist evaluation offers a valuable methodology for researchers investigating interventions utilising simulation-based education. By investigating and understanding the context, mechanisms and outcomes of SBE interventions, realist evaluation can provide the deeper level of understanding being called for.

## Introduction

The use of simulation devices in medical education is centuries old and includes anatomical models in the teaching of anatomy, threshold innovations such as Åsmund Lærdal’s Resusci Anne, modern high-fidelity manikins, simulated patients and virtual reality [1]. Simulation is defined as follows:

A technique that creates a situation or environment to allow persons to experience a representation of a real event for the purpose of practice, learning, evaluation, testing, or to gain understanding of systems or human actions [2].

Examining the features and use of simulation technology, the Best Evidence Medical Education (BEME) review of the literature from 1969 to 2003 [3], the authors concluded that the quality of published research for this period was generally weak. However, the available evidence suggested that high-fidelity simulations facilitate learning under the right conditions. A follow-up review of the literature from 2003 to 2009, using combined critical and realist review methodology, identified 12 features of best practice for simulation-based education (SBE) and concluded that simulation technology can produce substantial educational benefits [4]. Revisiting this review in 2016, McGaghie et al. [5] found that the evidence supporting SBE as a powerful educational intervention was growing.

In England, the Chief Medical Officer’s report for 2008 ‘Safer Medical Practice: Machines, manikins and Polo Mints’ states that, ‘Simulation offers an important route to safer care for patients’ and does so by improving performance, reducing errors and strengthening team work. The report recommends that simulation ‘needs to be more fully integrated into the health service’ [6]. This theme was further developed by Khan et al. [7] who built an argument for increasing expansion of SBE driven by patient safety and improvements in healthcare. They concluded that the continuing advances in simulation technology and an in-depth understanding of educational principles and practical applications of SBE to outcome-based programmes will help bridge the gap between the classroom and clinical environment.

This application of theoretical knowledge to the practical management of patients (the theory-practice gap) and the transition from student to doctor are key areas of interest in SBE research [8]. Reviewing the evidence for simulation to help bridge the perceived educational gap in students’ training and resolve the disconnect between classroom and clinical environment, Okuda et al. [9] found multiple studies that demonstrated the effectiveness of simulation in the teaching of basic science and clinical knowledge, procedural skills, teamwork and communication but only a few studies showing direct improvement in clinical outcomes. In summarising the outcomes of technology-enhanced simulation training for health profession learners, Cook et al. [10] found that compared to no intervention, technology-enhanced simulation is associated with large positive effects for knowledge, skills and behaviours and moderate effects for patient-related outcomes.

Reviewing the literature, Bell et al. [11] found a mixed picture as to the effectiveness of simulations as training tools and called for more theory-driven research focussed on the instructional capabilities of the technologies used in simulation. Echoing this call for more theory-driven research, Sevdalis [12] stressed the need for simulation studies to move away from presenting self-report data from small numbers of attendees to those that present a deeper theoretical and practical understanding of effective SBE. More recently, in the editorial marking the launch of *Advances in Simulation*, Nestel [13] reinforced the value of studies that deepen our understanding of SBE interventions and stated that ‘(when studying) more complex uses of simulation technologies, researchers have a responsibility to thoughtfully align research paradigms with hypotheses and research questions.’

This call for more theory-driven research is not confined to SBE. Cook et al. [14] proposed a framework to classify the purpose of educational research studies in four leading educational journals. The framework classified studies into one of three categories: description, justification and clarification. Their results showed that only 12% of reported articles could be classed as clarification studies with description at 16% and justification at 72%. Applying this framework to over 1300 abstracts from four major SBE conferences over 2 years (2014 and 2015), Graham et al. [15] found that only 9.3% of abstracts could be classified as clarification studies (description 54.4% and justification 36.3%).

There are a multitude of evaluation instruments and methods for the SBE researcher to choose from. In reviewing the published evaluation instruments for human patient simulation within nursing education, Kardong-Edgren et al. [16] found a lack of reliable and valid instruments to evaluate learning outcomes. They suggested a moratorium on ‘the indiscriminate development of new evaluation tools’ which focusses on self-reported satisfaction and confidence which could lead to the development of ‘a mile-wide and inch-deep evaluation landscape’. They recommend the use of multiple instruments to evaluate a simulation in order to capture all the learning domains and to explore how actions in the simulations carry over into the clinical arena. However, the evaluation instruments reviewed did not address the issue of how or why the interventions being studied achieved their outcomes.

Whether an intervention is successful or not was highlighted by Ogrinc and Batalden [17] who argued that traditional study designs such as randomised controlled trials, nonrandomised and prospective cohort studies while useful, depending on the focus of the evaluation, fell short in a key component, namely being able to identify the depth of contextual information that is helpful when replicating the findings in another setting. One such study design is the Context, Input, Process and Product evaluation (CIPP) model [18] which seeks to answer four kinds of questions. These are ‘What should we do?’, ‘How should we do it?’, ‘Are we doing it correctly?’ and ‘Did it work?’ However, it does not specifically address the questions ‘How and Why the intervention worked?’; the answers to which are required to provide a deeper theoretical and practical understanding of effective SBE [12, 13]. One evaluation methodology that explores both the context and underlying mechanisms of how and why a programme works (or not) is realist (realistic) evaluation [19].

## Realist evaluation

Realism is a philosophy which positions itself between positivism and constructivism. Positivism describes reality as fixed and our knowledge of that reality, which is neutral/value free, can be described by theories that are objective and generalizable. Positivist research aims to discover what exists through prediction and control using mainly quantitative methods with the researcher being an independent observer [20]. Conversely, constructivism views reality and knowledge of that reality as not fixed but socially constructed and this knowledge has both multiple constructions and values. Constructivist researchers are active participants in the research and use both quantitative and qualitative methods [21]. The realist view of knowledge is that there is a real world, and through our senses, brains and culture, we process our knowledge of it [22]. In relating this to the clinical environment, there is a real world of patients, signs and symptoms (positivism) and these are open to a variety of interpretations which depend on the complex interaction of external influences on the clinician (constructivism).

Realist evaluation seeks to answer the question ‘What works for whom, in what circumstances and why?’ [23]. In answering this question, the realist researcher seeks to identify, test and refine the components of an educational programme that work as well as those that do not. The three fundamental components that realist evaluation seeks to investigate are context, mechanism and outcome. In other words, educational programmes work (successful outcome) when they provide appropriate opportunities or resources (mechanisms) to groups under appropriate conditions (context). This is known as the ‘context-mechanism-outcome (CMO) configuration’ [19] and can be written as the formula *context + mechanism = outcome*. There is no set limit on the number of proposed CMO configurations that are constructed for the educational programme under investigation; the key element is the relationship within each CMO [17]. The researcher gathers data in order to test the proposed CMO configurations.

Simulation is a complex educational intervention with multiple interacting components which can make it challenging to evaluate. However, realist evaluation may provide more useful information about its effectiveness than traditional models of education evaluation [24]. So how might a realist evaluation of an SBE programme be designed? The guiding framework is the realist evaluation cycle which has four key steps [19].

- Step 1. Formulate a working theory. One of the key areas of interest within SBE is the impact of medical school simulation-based learning on newly qualified doctors’ performance [8]. Exploring this example, the working theory would be ‘A one-day simulation-based course would enhance the transition from final year medical student to newly qualified doctor’.
- Step 2. Hypothesis. Formulate the hypothetical CMO configurations, i.e. what *might* work for whom in what circumstances and why? Table 1 presents an example of the proposed CMO configurations for the SBE course aimed at final year medical students.
- Step 3. Observations. Test the theory by gathering data on the CMO configurations using a mixed methods approach (quantitative and qualitative data collection and analysis). The researcher is not limited to a particular method but can choose whichever approach to collecting and analysing the data that suits the intervention under study.
- Step 4. Programme specification. Reveals what *did* work for whom in what circumstances and why. This provides a refined theory which will inform future interventions and programme evaluations. The process then continues in an iterative cycle.

**Table 1:** Proposed CMO configurations for the SBE course for final year medical students

Context	Mechanism	Outcome (students)
C1. Final year medical students in their last few months of training.	M1. Providing the opportunity to experience and explore the role of a newly qualified doctor in a simulated setting.	O1. To foster an understanding of the role of a newly qualified doctor.
C2. Final year medical students who have extensive knowledge and clinical experience but have not had the responsibility of managing the acutely unwell patient.	M2. Presenting a variety of realistic simulated medical and surgical emergencies using a high-fidelity manikin.	O2. To assess and manage the acutely unwell patient using a structured approach.
C3. The majority of students have observed the management of a cardiac arrest.	M3. Allowing the students to manage (as a team) a simulated cardiac arrest.	O3. To increase understanding of team work and communication.
C4. Before commencing as newly qualified doctors the students undertake a 6-week assistantship.	M4. Exploring the role of the newly qualified doctor and setting goals for assistantship.	O4. To identify and set goals for assistantship.
C5. The students have varying levels of confidence.	M5. Providing immediate feedback and exploring the factors that influence when and why the students call for assistance.	O5. To recognise personal limitations and when to call for help.

### Context

The context reflects the reality into which an intervention is introduced and provides the conditions (Table 1) that trigger the mechanisms to produce the desired outcomes [19] and requires that all elements that are relevant to the mechanisms be considered [17]. Just as all social programmes are introduced into pre-existing social contexts, so SBE programmes are introduced into pre-existing healthcare and or educational contexts. Therefore, researchers should not ignore the contexts of their programmes and to do so is regarded by Pawson and Tilley as one of the ‘great omissions of evaluation research’ [19].

In their critical review of simulation-based research: 2003–2009, McGaghie et al. [4] highlighted 12 features and best practice of SBE that teachers should know in order to use simulation technology to maximise educational benefit. A number of these related to context and included how the intervention integrated into the wider medical curriculum and its outcomes, simulation fidelity, instructor training and the educational and professional context within which the interventions occurred. However, they cautioned that the introduction of a complex service intervention, such as SBE, into medical education environments would not be easy and with time may re-shape the goals and practices of those same educational programmes, thus changing the original context [4].

Acknowledging the importance of context and prompted by the recognition that SBE has several unique features including a wide variety of simulation modalities and instructional design, Cheng et al. [25] called for those reporting simulation-based research to provide more detailed descriptions of the context within which their interventions occurred. The key elements to report are participant orientation, simulator type, simulator environment, simulation event/scenario, instructional design or exposure and method of feedback/debriefing.

While some of these contextual elements are easily described and can, to a limited degree, be standardised for research purposes, e.g. fidelity of simulator and scenario design [26] others are not. In our experience, these elements usually relate to the students and how they construct their own version of the ‘contextual reality’ as they interact with the faculty, each other and the environment [27]. This interplay between individuals and the educational programme means that the causal mechanisms are located in the social relations and context as well as the individuals [28].

Drawing on the experience of a realist evaluation of a simulation-based course for final year medical students (unpublished) conducted as part of a higher degree (ACG) examples of contextual elements that can affect learning and may not be easily identified through other evaluation approaches includes students who have significant anxieties about SBE, those delegates ‘forced’ to attend by their line managers, inadequate orientation to the simulated environment, instructor training and experience and the timing of the course in relation to other significant events, e.g. final examinations.

### Mechanism

Explanatory mechanisms are the central tenet of realist evaluation and comprise the processes/resources and responses (reasoning) of stakeholders to those processes/resources, operating in a given context, that generate the outcomes of a programme [19]. Mechanisms can be ‘visible’ and form part of the design of an evaluation or ‘invisible’ and only come to light during the evaluation process [23].

Mechanisms are said to fire or be triggered in a given context to create an outcome. Pawson and Tilley explain this using the gunpowder analogy [19] in which the chemical composition of the gunpowder is the mechanism that creates an explosion (outcome) when a spark is applied. However, if the conditions (context) are not favourable, e.g. damp gunpowder or no oxygen present, then there is no explosion. This ‘on/off’ response has been challenged by Dalkin et al. [29] who argue that activation of a mechanism operates on a continuum similar to a light dimmer switch. They believe that this has more explanatory value in understanding how interventions work leading to a graduated response of outcomes and fits with our experience where learning outcomes do not usually operate on a met/not met basis, e.g. unskilled/completely skilled or no confidence/complete confidence.

In helping to clarify the concept of mechanism, Astbury and Leeuw [30] highlight what mechanisms are not. Firstly, evaluators should make a clear distinction between mechanism and programme activity. For example, it is not an SBE intervention in and of itself that generates the outcomes but the knowledge gained or the increase in confidence of the participants. Secondly, mechanisms should not be considered as independent causal variables; rather, they attempt to explain why variables are related. That is, how did the SBE intervention cause an increase in participant confidence and how did this generate the observed outcomes.

Another challenge for the realist evaluator is to distinguish between context and mechanism. To help differentiate between the two, Dalkin et al. [29] proposed an alternative operationalization of Pawson and Tilley’s formula, *context + mechanism = outcome* [19], which explicitly disaggregates mechanism into its component parts; the resources offered and the changes in reasoning of the participants. The new formula is written as *M (resources) + context → M (reasoning) = outcome* and provides both an operational and conceptual clarification of mechanism [29]. For example, Cheng et al. [25] list ‘simulator type’ as a contextual element; however, applying the revised formula what was previously considered contextual becomes part of the mechanism. Simulators vary in type and level of ‘fidelity’ and their effective use depends on matching the educational goals to the simulation tools used and taking into account the level of expertise of the participants [4]. As a result, the ‘simulator type’ becomes the M (resource), the participants’ level of expertise is the context, how the participants interact with and learn from the simulator is M (reasoning) and the outcome is the measurable change in the participants’ skill and/or knowledge.

When considering the concept of mechanism (resources and reasoning), educators should be cognisant of the educational theories/conceptual frameworks that underpin the resources they offer as well as the change in reasoning that may occur as a result and should declare these in their evaluations. So what are some of the educational theories/conceptual frameworks that underpin SBE? In their realist review, McGaghie et al. [4] identified the following: feedback, deliberate practice, mastery learning, team training and high-stake testing (can also be considered an outcome) while Ker and Bradley [31] highlighted social constructivism, experiential learning, reflective learning and activity theory. More recently, Graham et al. [15] reported the ten most commonly declared educational theories/conceptual frameworks in abstracts from simulation conferences. These were, in descending order, cognitive theories, experiential learning, gaming theories, learning styles, deliberate practice, inter-professional learning, mastery learning, realism, self-regulated learning and the flipped classroom. Table 1 shows the proposed mechanisms for the simulation-based course for final year medical students. Using mechanisms M2 and M4 as examples, we can explore the M (resource), M (reasoning) and educational theory for each.

M2. The M (resource) is the high-fidelity manikin chosen because the students have extensive clinical experience (context) and so expect to elicit realistic signs and symptoms from the manikin as well as have it react in real time during the scenario. The M (reasoning) is the students recognising the value of a structured approach to managing the acutely unwell patient while putting theory into practice. The underlying conceptual framework is activity theory which states that learning, knowledge and activity are intrinsically linked and there is a relationship between one activity system and another, in this case, the simulated and the clinical environments [31]. It also stresses the concept of contradiction and tension in learning [32] which in this example is between the students’ theoretical knowledge of how to manage the acutely unwell patient and their practical ability to do so. The desired outcome is the students become more proficient in using a structured approach when assessing and managing the acutely unwell patient.

M4. The M (resource) is giving the students the opportunity to manage the scenarios as if they were the newly qualified doctor on the ward, and the context is the impending 6 week assistantship. The M (reasoning) is by allowing the students to explore the roles and responsibilities highlighted by the scenario, they would set personalised goals for the assistantship (outcome). The underlying conceptual framework is self-regulation theory which seeks to optimise learning and performance using goal-directed behaviour [33].

This list is not exhaustive, and each researcher should identify the key mechanisms they consider to be operating within their own SBE programme that are thought to produce the desired/measured outcomes.

### Outcome and data collection

Outcomes of educational interventions can be expected (mastery of a skill), unexpected (collateral effects on the participants or their place of work), positive (an increase in knowledge) or negative (psychological harm from a poorly conducted debrief session). In addition, programme outcomes cannot just be viewed as undifferentiated wholes but rather as the complex outworking of multiple mechanism/context effects [19]. There are a number of approaches available when describing and evaluating the outcomes of educational programmes.

Bloom’s taxonomy [34] classifies the learning outcomes that educators set for their educational programme into three domains: cognitive, affective and psychomotor. Using this framework, the outcomes for the simulation-based course for the final year medical students (Table 1) are cognitive—to foster an understanding of the role of a newly qualified doctor and to increase understanding of team work and communication, affective—to recognise personal limitations and when to call for help and psychomotor—to assess and manage the acutely unwell patient using a structured approach and to identify and set goals for assistantship.

Kirkpatrick’s hierarchy [35] is one of the most widely applied approaches and describes the value and worth of training. It has four levels, with the evidence for higher levels being harder to collect: (level 1) reaction—how do the participants react favourably (or not) to the event, (level 2) learning—what knowledge, skills and attitudes do the participants acquire as a result of the event, (level 3) behaviour—to what degree do the participants apply what they have learned during the event and (level 4) results—what targeted outcomes occur as a result of the event at an organisational level, e.g. improved patient outcomes.

Another approach is the use of translational science outcomes which has been highlighted as useful for SBE research [8, 36, 37]. There are four levels which are said to move from ‘the bench to the bedside [8]’. These are (T1) educational effects achieved in educational laboratories, (T2) improved patient care practices, (T3) better patient outcomes and (T4) collateral educational effects.

Realist evaluation uses a mixed methods approach to data collection [19] which involves the collection, analysis and interpretation of both quantitative and qualitative data in a single study [38]. This has been shown to be of benefit when studying complex interactions [39]. The triangulation of data from different sources allows for a richer and fuller explanation of the data [40], and the evaluation takes the form of an iterative explanation-building process [41]. This methodological diversity has been recognised as an important development within medical education [5]. The aim of the realist researcher is to understand the patterns of outcomes that result from the firing of different mechanisms in different contexts and the relationship between them [17].

Taking the evaluation of the simulation-based course for final year medical students as an example, a routine course evaluation questionnaire using a 5-point Likert scale [42] completed immediately after the course would provide Kirkpatrick level 1 data about the students’ satisfaction with the course, the effectiveness of the debriefing, relevance to their work, length and timing of the course. Kirkpatrick level 2 and 3 data could be obtained from a follow-up questionnaire, with space for free text, sent out after the students complete their first rotation as newly qualified doctors that investigates what lessons had been learned from the course and whether these had been applied in their new role. Further qualitative data can be obtained from individual interviews or focus groups [40] which explore the proposed CMO configurations. That is, the effect of context, the proposed enabling mechanisms and the extent to which the outcomes had been achieved and if not, why not? Patient outcome and quality of care data are more challenging to collect requiring the researcher to identify or construct suitable databases that can be used to study the outcomes at an organisational level [8].

## Discussion

Using a methodology that clarifies why an intervention works (or not) by examining all of its component parts, context, mechanism and outcome, allows others to better interpret the results, deepens understanding and helps to advance SBE research [8, 12, 13]. In our unpublished evaluation, we discovered that although the students reported that the course had helped with the transition from student to newly qualified doctor, not all of the students were setting goals for their assistantship. The focus group data revealed that the timing of the course (context) before the final examinations meant that the students’ priorities were the exams and not setting goals for the assistantship. Thus, the context prevented the mechanism (exploring the role of the newly qualified doctor and goal setting) from firing which adversely affected the desired outcome.

Pawson, by his own admission, does not claim that realist evaluation is perfect and mentions a number of difficulties that arise when trying to apply realist principles [23]. These include the absence of an explanatory focus, using only one data collection method, failure to investigate the CMO configuration and the restrictive word counts imposed by some publications. From our own experience, the practical challenges included a poor response rate (26.3%) to the follow-up questionnaire, no one turning up to one of the arranged focus groups and too many turning up to another potentially inhibiting some of the quieter members of the group.

So what has realist evaluation delivered so far in the field of healthcare? In their review of realist evaluations, Marchal et al. [28] found 18 papers describing realist evaluations across a variety of healthcare settings. They showed that the uptake of realist methodology has been slow; however, they argue that even a superficial application of realist evaluation has advantages as it explores the processes and context rather than just the intervention and its outcomes (did it work?). They admit that more clarity is needed concerning how the terms—context and mechanisms—are defined and call for more conceptual work to allow a greater understanding of these issues.

Krupat [43] has called for research that is conceptual and thoughtful and identifies the mechanisms that mediate and moderate the relationship between action and outcome. Exploring and developing theories about mechanisms can add value to programme evaluation by revealing why a programme works which in turn can better inform the design and evaluation of future programmes [30]. Regehr has highlighted the need for health profession education research to re-orientate its alignment with the imperative of proof to one of understanding, and from the imperative of simplicity to one of representing complexity well [44]. Realist evaluation is a methodology that is able to address these issues by exploring all aspects of an intervention. There are challenges in performing realist evaluations, but we encourage the simulation community to adopt its principles and by doing so help clarify and define the contexts, mechanisms and outcomes that are unique to our ‘simulated version of reality’ and help answer ‘What works for whom in what circumstances and why?’ [23].

## Abbreviations

BEME: Best Evidence Medical Education
CIPP: Context, Input, Process and Product
CMO: Context, mechanism and outcome
SBE: Simulation-based education
